# Sex Differences in Orienting to Pictures with and without Humans: Evidence from the Cardiac Evoked Response (ECR) and the Cortical Long Latency Parietal Positivity (LPP)

**DOI:** 10.1371/journal.pone.0108224

**Published:** 2014-10-15

**Authors:** Monika Althaus, Yvonne Groen, Lutske van der Schaft, Ruud B. Minderaa, Oliver Tucha, Lambertus J. M. Mulder, Albertus A. Wijers

**Affiliations:** 1 Department of Child- and Adolescent Psychiatry, University Medical Center Groningen, Groningen, The Netherlands; 2 Department of Psychology, University of Groningen, Groningen, The Netherlands; Vanderbilt University, United States of America

## Abstract

**Objective:**

This study investigated the effect of social relevance in affective pictures on two orienting responses, i.e. the evoked cardiac response (ECR), and a long latency cortical evoked potential (LPP) and whether this effect would differ between males and females. Assuming that orienting to affective social information is fundamental to experiencing affective empathy, associations between self-report measures of empathy and the two orienting responses were investigated.

**Method:**

ECRs were obtained from 34 female and 30 male students, and LPPs from 25 female and 27 male students viewing 414 pictures from the International Affective Picture System. Pictures portrayed pleasant, unpleasant and neutral scenes with and without humans.

**Results:**

Both the ECR and LPP showed the largest response to pictures with humans in unpleasant situations. For both measures, the responses to pictures with humans correlated with self-report measures of empathy. While we found a greater male than female responsiveness to the pictures without humans in the ECR, a greater female than male responsiveness was observed in the LPP response to pictures with humans.

**Conclusion and Significance:**

The sensitivity of these orienting responses to social relevance and their differential contribution to the prediction of individual differences underline the validity of their combined use in clinical studies investigating individuals with social disabilities.

## Introduction

### 1. Orienting and motivational activation

Orienting to significant environmental input reflects the engagement of two motivational systems, a defensive and an appetitive one, which have evolved to ensure the survival of the individual and his progeny [1], [2]. These systems are supposed to be implemented by limbic structures in the brain and to be broadly similar across the mammalian species. Activation of the appetitive system should occur in response to stimuli experienced as attractive and potentially life sustaining, i.e. stimuli with a pleasant hedonic valence leading to approach behavior, while activation of the defense system should occur in response to potentially threatening, aversive and unpleasant stimuli leading to avoidance or attack.

Orienting has moreover been conceptualized as a set of functional physiological responses with different habituation rates that should index specific central, perceptual and motor processes [1]. Among the responses investigated with respect to their sensitivity to stimulus novelty, stimulus significance and exposure time were heart rate, i.e. the evoked cardiac response (ECR) and a long latency positive EEG event-related potential (ERP) component, the LPP. These responses were extensively studied during the passive watching of pleasant, unpleasant and neutral pictures selected from the International Affective Picture System (IAPS) [3]. Concerning heart rate, a cardiac deceleration response was found consisting of a fast initial non-habituating bradycardia, previously conceptualized as reflecting a “transient detection response” or “stimulus registration process” [4] followed by a prolonged somewhat slower deceleration that was shown to be sensitive to the emotional valence of stimuli, yet only in case the stimuli were novel. This second deceleration component has been interpreted as being indicative of a reflexively enhanced orienting response, which has been found particularly for aversive cues [1]. No valence-differential responses were observed when exposure time was short (≤500 ms) supporting the previously proposed hypothesis that the cardiac deceleration response is related to sensory intake processes [5], [6], especially when extensive perceptual processing is required. Different from the ECR, the LPP appeared (1) not to habituate (not even after 90 repetitions of the same stimulus within the same experimental session), (2) to show valence-dependent enhancement even when stimuli are presented as short as 25 ms, and (3) to persist for seconds after picture presentation [7]. This led to the suggestion that the LPP “is a persistent sign that the evoking stimulus is motivationally relevant” [2] and that it could be viewed as a central correlate for cognitive evaluation of stimulus significance [1].

In the paper by Bradley [1], significance of the environmental input was defined in terms of its hedonic valence and arousal value. In the present study, another dimension was added to the aspect of significance. This dimension refers to the social relevance of information which has only recently begun to be studied systematically. Using the same passive picture viewing paradigm as in the studies by Bradley and colleagues [3], [5], both Proverbio and colleagues [8] and Groen and colleagues [9] investigated the (interactive) effects of social relevance and hedonic valence on ERPs by presenting affective pictures with and without humans. Pictures with humans depicted negative emotions such as pain, anger, fear, and suffering or positive emotions such as happiness, love, and tenderness. The main focus of both studies was the investigation of sex differences in ERPs related to the processing of affective scenes with and without humans. The study from our group [9] was a replication and extension of the study by Proverbio and colleagues [8]. The extension consisted of (1) adding emotionally neutral pictures to allow measuring non-emotional stimulus processing, (2) removing the most disgusting pictures from the selection, (3) recording heart rate, and (4) assessing self-reported empathic traits. Questionnaires of empathy were included to investigate brain-behavior correlations based on the assumption that experiencing affective empathy is contingent upon enhanced neurophysiological responding to watching people in emotionally exciting situations. The present paper uses data obtained in the study by Groen et al. [9]. Extending on our previous paper, we here focus on a comparison of cardiac and brain potential measures, i.e. on the ECR and the LPP that have both been associated with orienting to significant input from the environment.

### 2. Sex Differences in emotional reactivity and empathy

Several studies using psychophysiological indices have reported sex differences in emotional reactivity with women generally showing stronger physiological reactions to especially negative, aversive pictures. This was expressed by, for example, greater decelerative cardiac responses, more facial EMG activity [10], and a prolonged recovery time as reflected by a continued potentiation of the startle reflex after negative picture presentation [11]. These findings have been confirmed by several functional magnetic resonance imaging (fMRI) studies [12] leading to the suggestion that women bear greater reactivity of the aversive motivational or defensive behavior inhibition system, while, moreover, men might show greater reactivity of the appetitive motivational or behavioral approach system. In the studies in which men were found to show greater responsiveness to positive pictures, the set of positive images contained, however, erotic pictures which had previously been shown to be rated as more pleasant and arousing by men than by women [10]. To prevent this potentially confounding effect, erotic pictures were excluded in the study by Proverbio and colleagues, Groen and colleagues and hence in the present study.

Summarizing the findings from the ERP studies by Proverbio and colleagues and Groen and colleagues, the presence of humans in pictures had, across both sexes, an effect on both early and late ERP components. ERPs were larger in response to photographs with humans depicted. For the early components, this held in particular for the responses to pleasant pictures, while the LPP showed the largest response to human pictures with unpleasant emotions. These findings suggested that positive emotions conveyed by humans are extracted from stimuli at early information processing stages, while negative emotions conveyed by humans get a greater significance at a later stage. Concerning sex differences, women showed a larger response to pictures with humans in both early ERPs and the LPP. In the Groen et al. study, this was, however, independent of the pictures’ emotional valence. Women moreover showed a larger response to unpleasant pictures, as was particularly evidenced by the LPP. Although the female response to unpleasant pictures seemed to be larger especially for pictures with humans, the three-way interaction between sex, valence and the presence of humans in the pictures failed significance. In contrast to the study by Proverbio and colleagues, the study by Groen and colleagues therefore did not provide electrophysiological evidence for a female enhanced processing of pictures with humans showing unpleasant emotions or being in unpleasant situations.

Furthermore, a series of studies has demonstrated sex differences with females scoring higher on self-report questionnaires measuring empathy [13]–[15]. A commonly made distinction is that between *affective* and *cognitive empathy* [13], [16]. Affective empathy refers to experiencing or sensing an emotion that is triggered by observing the emotion of another person (emotional contagion), whereas cognitive empathy refers to understanding and evaluating the feelings of others while attributing a mental state to the other person (affective mentalizing). In the study by Groen and colleagues, females were found to report significantly more affective empathy than males, while no sex difference was found on the specific measure of cognitive empathy. Moreover, both short- and long-latency ERP responses to human-specific emotions were associated with self-reports of affective but not cognitive empathy.

Another distinction described to discriminate males from females is the one that refers to *empathizing* versus *systemizing* behavior [13], [17], with empathizing being defined as the drive to identify another person’s emotions and thoughts, and to respond to these with an appropriate emotion, and systemizing as the drive to identify the rules that govern a system in order to predict how this system will behave [18]. Several self-report studies have demonstrated that more males than females have a “systemizing (S) brain”, and that more females than males have an “empathizing (E) brain” [13], [19].To our best knowledge this distinction has not yet been related to sex differences in orienting responses to affective stimuli before and will therefore be explored in the present study.

### 3. Orienting responses to affective pictures with humans

Given that heart rate slowing has repeatedly been found in response to visual affective stimuli with decelerations generally being steeper in response to negative as compared to neutral and positive stimuli [20]–[24], we were interested in the question whether the cardiac deceleration response, just like the LPP, would be sensitive to the social information in the pictures as well. Phasic decelerative changes in heart rate are mainly under the control of parasympathetic (vagal) nervous processes [25], [26] with the size of the deceleration being suggested not only to depend on the valence but also on the relevance of a stimulus [27], [28]. Next to being associated with extensive sensory intake processes (see above), these changes have been described as a vagally mediated *anticipatory* response preparing the organism for some effective motor performance [29]. Slowing of the heart beat provides more time for the heart to fill such that at the time a motor response is required the organism’s metabolic demands are more efficiently met by a better supply of oxygen and nutrients to the muscles. If this motor preparatory cardiac response turns out to be more sensitive to affective pictures with humans than to affective pictures without humans, this would suggest the cardiac orienting response to be sensitive to the motivational significance of affective social information. Moreover, if this response differs for men and women, the presence of humans might have a different motivationally activating meaning for the two sexes. As the anticipatory role of heart rate slowing might be functionally different from what the LPP has been proposed to reflect, i.e. cognitive evaluation of the stimulus relevance, the two responses to affective social situations might differ, thereby reflecting different aspects of orienting.

### 4. Aims and Expectations

The primary aim of the present study was to compare the cardiac evoked orienting response and the cortical evoked LPP response with each other in terms of their sensitivity to social relevance, hedonic valence and sex effects. We hypothesized that if the cardiac deceleration response is sensitive to the social relevance of the pictures, (1) an effect of humans in the pictures should be found, and (2) that this effect should be most prominent for the aversive pictures. Confirmation of these two hypotheses would agree with what has previously been found for the LPP. As women have been reported both to be more empathic and to show greater physiological responsiveness to aversive stimuli, (3) a greater heart rate response to aversive pictures with humans should be found in women than in men. Confirmation of this hypothesis would agree with what has previously been found for the LPP by Proverbio and colleagues [8], but disagree with the findings on the LPP by our own group [9]. In this context we further explored whether the two responses would complimentarily predict sex differences.

A secondary aim of the study was to explore whether the two orienting responses to affective pictures with humans are associated with self-report measures of empathy-related traits. Based on the assumption that experiencing affective empathy (emotional contagion) is contingent upon enhanced neurophysiological responding to watching people in emotionally exciting situations, we expected that both orienting responses would be associated with measures of in particular affective empathy.

## Methods

### Ethics statement

The present study was conducted according to the guidelines for ethical conduct and report of research. The study had been approved by the Ethics Committee of Psychology of the University of Groningen and written informed consent was obtained from all participants.

### 1. Participants

Sixty-seven healthy psychology students (35 women), in the first year of their study, 18–28 years of age (M = 20.8 years; SD = 2.0 years) participated in the experiment while earning academic credits. Participants were unaware of the exact goal of the study and were told that sex differences in emotion processing were investigated. After participation, the participants were debriefed. Presence of psychopathology was checked by means of the 90-item Symptom Checklist (SCL-90) [30]. Based on its total scale score measuring “psychoneuroticism” two men and one woman were found to score in its clinical range; these were therefore left out of the study. The remaining 30 men and 34 women did not significantly differ in their SCL-90 total score (males: *M* (*SD*) = 116.50 (20.5); females: *M* (*SD*) = 120.61 (21.52); *t* = 0.78; *p* = .43; *d* = 0.19). The males, however, were on average one year older than the females (males: *M* (*SD*) = 21.07 (2.1) years; females *M* (*SD*) = 20.03 (1.9) years; *t* = 2.07; *p* = .04; *d* = 0.63).

Participants completed Dutch translations of two questionnaires consisting of respectively 60 and 75 items, which were used to obtain an “Empathizing Quotient” (EQ), and a “Systemizing Quotient” (SQ) [13], [17]. Following Wheelwright and colleagues [18], we computed a standardized discrepancy score (SQ-EQ) with higher scores indicating a more systemizing than empathizing way of processing information from the environment. Participants also completed a Dutch translation of the Interpersonal Reactivity Index [14]. The IRI contains four subscales with seven items each. The Perspective Taking (PT) scale describes the tendency to adopt the psychological viewpoint of others; the Empathic Concern (EC) scale assesses feelings of compassion and sympathy for others; the Fantasy subscale (FS) examines the propensity to take the view of others in fictional situations; and the Personal Distress (PD) scale measures the tendency to experience emotional distress in response to perceived distress in others. The PT and FS subscales are assumed to measure cognitive empathy, while the EC and PD subscales are assumed to assess emotional (affective) empathy. Internal scale consistencies as measured by Cronbach’s alpha are reported in Table 1.

**Table 1:** Mean values and standard deviations for the self report measures.

		Males		Females				
	α*	Mean	SD	Mean	SD	t	p	d
EQ	.79	44.27	8.94	51.26	6.62	−3.58	**.001**	0.91
SQ	.89	59.08	13.07	42.44	16.56	3.84	**<.001**	0.98
SQ-EQ	-	0.092	0.06	−0.004	0.06	5.23	**<.001**	1.51
IRI_Perspective Taking	.77	18.96	2.94	18.67	3.60	0.35	.728	0.09
IRI_Empathic Concern	.77	17.07	3.99	19.29	3.43	−2.4	**.019**	0.61
IRI_Fantasy	.66	15.30	5.66	17.97	3.89	−2.19	**.030**	0.56
IRI_Personal Distress	.80	8.800	3.91	12.65	4.27	−3.74	**<.001**	0.95

T-test results are reported for sex differences.

*Cronbach’s α based on our sample of respectively n = 64 for the EQ and IRI-scales and n = 48 for the SQ questionnaire.

Data of the EQ and IRI were obtained from all the 64 subjects participating in the study, while the data of the SQ were available from only 48 of them (25 females), because this questionnaire was introduced at a later stage of the study. Table 1 shows that, on a test-wise level, for all but one of the questionnaire scales, significant sex differences were found with higher scores for women except for the SQ, which was significantly higher for men. Men and women did not differ in their scores on Perspective Taking.

### 2. Task and dependent measures

#### 2.1 IAPS Images

The experiment consisted of the presentation of 414 IAPS pictures that were equally distributed across six categories representing respectively emotionally neutral, positive and negative images with or without humans being portrayed. Of these pictures, 69 depicted emotionally neutral humans (e.g., neutrally looking people), 69 positive emotional humans (e.g. happy, soothing and smiling people), 69 negative emotional humans (e.g. frightened, crying and pain suffering people), 69 neutral scenes (e.g. household objects, plants and neutral animals), 69 positive emotional scenes (e.g. beautiful environments and natural events, friendly animals), and 69 negative emotional scenes (e.g. dirty environments, aggressive animals, natural catastrophes). In agreement with Proverbio and colleagues (2009), erotic pictures were left out from the set of positive pictures, because these had previously been shown to be rated as more pleasant and arousing by men than by women [10]. Different from the Proverbio study, the most disgusting pictures were excluded as well in order to prevent a bias towards feelings of disgust. The following pictures were included in the experiment. **Human Positive**: 2332, 2339, 2057, 2154, 2160, 2510, 2170, 2331, 2152, 4603, 2037, 2370, 4532, 8280, 4606, 5410, 4623, 8205, 2224, 2165, 4533, 8185, 4689, 8496, 2391, 2598, 2389, 2373, 2058, 4220, 8031, 1601, 4700, 2660, 2030, 8040, 4610, 7325, 2299, 8032, 8193, 2091, 8250, 8200, 4614, 2260, 2501, 5470, 8041, 2040, 2222, 5621, 2655, 4542, 2387, 8080, 8186, 4250, 2550, 1340, 8034, 5831, 2311, 2092, 2360, 2530, 8033, 4641, 2388. **Human Negative**: 3017, 3191, 3215, 6350, 2717, 3301, 2799, 6510, 6360, 8230, 9810, 2375, 9428, 9530, 3016, 2750, 3180, 3266, 9254, 3550, 9592, 2800, 2141, 9800, 3181, 3140, 9429, 2095, 2900, 3069, 3068, 9421, 6834, 6313, 6315, 3170, 2205, 9400, 2710, 3101, 3300, 2053, 9903, 3071, 9220, 3530, 2661, 9635, 2730, 3080, 9252, 3500, 2352, 9520, 3030, 9420, 3051, 3130, 3060, 3100, 9410, 9415, 9265, 3053, 3064, 8485, 9253, 3015, 9423. **Human Neutral**: 2104, 2605, 2397, 2570, 2381, 2440, 2320, 2200, 2230, 2480, 2271, 2210, 2516, 2518, 2270, 2038, 2580, 2372, 2495, 2221, 2493, 5410, 2102, 2485, 2500, 2394, 2620, 2410, 2385, 2850, 2190, 2250, 2520, 2512, 2514, 2396, 2595, 2499, 2357, 2575, 7620, 2305, 2383, 2220, 2515, 2579, 2499, 2357, 2575, 7620, 2305, 2383, 2220, 2515, 2579, 2749, 2594, 2393, 2560, 7496, 2980, 7493, 2272, 2191, 5875, 2235, 2388, 2593, 2745. **Scenes Positive**: 1441, 7192, 1450, 1675, 5594, 5551, 5814, 1661, 7330, 7220, 5611, 5820, 7057, 1604, 1440, 5890, 5891, 1811, 5870, 5450, 1750, 1560, 5030, 7260, 1602, 5593, 1590, 7480, 1910, 1710, 5000, 5600, 1810, 7039, 1419, 5910, 7320, 1670, 7291, 7430, 5711, 1720, 1812, 5020, 5010, 1463, 1900, 1333, 7472, 5849, 7450, 1920, 5200, 1610, 5480, 1721, 5780, 5779, 1603, 1660, 1460, 5720, 5001, 5750, 5731, 5760, 5201, 1600, 7242. **Scenes Negative**: 5961, 1050, 9630, 1113, 5972, 1321, 1051, 1525, 9925, 6415, 1617, 1120, 9901, 9500, 9090, 9300, 9360, 1022, 5920, 9080, 5971, 9301, 5950, 9622, 7054, 9830, 1026, 9180, 9635, 5940, 1310, 1300, 9495, 6800, 1101, 9560, 7110, 9912, 1931, 1040, 9561, 9571, 9290, 6610, 1932, 9600, 6930, 1280, 9008, 1090, 9920, 9401, 7920, 9181, 9373, 1019, 9001, 1114, 1110, 9902, 9911, 9340, 1030, 1111, 9342, 9409, 9171, 9000, 9182. **Scenes Neutral**: 7058, 7043, 7053, 5631, 7180, 7546, 7545, 7046, 7031, 7059, 5594, 7052, 2445, 7060, 7044, 7217, 5635, 7224, 5471, 7547, 7150, 7504, 7175, 7235, 7096, 7590, 7170, 5611, 2206, 6150, 7095, 5510, 7000, 2446, 7006, 7035, 7234, 7009, 7054, 7503, 7050, 7140, 5500, 1810, 7161, 7211, 5530, 7039, 7010, 1670, 7090, 5740, 7034, 7510, 7004, 5900, 7233, 5520, 7037, 5531, 7080, 5661, 7002, 5532, 5130, 7036, 5120, 7038, 7179.

The IAPS is a well-established and widely used source of images eliciting affective responses [3]. It consists of 942 color photographs and includes gender and age dependent normative ratings of the images on dimensions that refer to feelings of pleasantness (valence) and excitement (arousal). Wondering whether the affective pictures with and without humans should not be matched on these subjective ratings of intensity (valence) and/or arousal, we did some analyses on the normative ratings previously obtained from samples of men and women [3] for the various pictures we selected for the 6 categories (see Table 2). We first of all found interactive effects of the presence vs. absence of humans with the pictures’ emotional valence on both the subjective ratings of valence (*F*(2,408) = 46.66; *p*<.001; *η^2^* = .186) and arousal (*F*(2,408) = 2.94; *p* = .05; *η^2^* = .014). Although there were significant differences in arousal and valence ratings between pictures with and without humans for the positive and neutral pictures as well, for the negative pictures these differences were significantly greater. Concerning arousal, this completely agrees with a finding by Norman and colleagues [31] who used a smaller collection of IAPS pictures with pleasant and unpleasant pictures being matched on normative ratings of arousal and valence intensity. The presence of humans in unpleasant pictures obviously reinforces the subjective feeling of unpleasantness and arousal. We moreover found that, independent of whether humans were depicted, our selection of negative images had previously been rated as significantly less pleasant by women than by men (*p*<.001; *d* = 0.86) and that the positive pictures were rated as significantly more pleasant by women than by men (p<.001; *d* = 0.86). Our selection of negative pictures had moreover been experienced as more arousing by women than by men (*p*<.001), with medium and small effect sizes for pictures with humans (*d* = 0.69) and without humans (*d* = 0.30), respectively. Independent of valence, both men and women had rated the pictures with humans as more arousing than the pictures without humans (men: *p* = .001; *d* = .33; women: *p*<.001; *d* = .47). We further investigated the correlations between valence and arousal ratings and whether these would differ for pictures with and without humans. We did find them to differ, and these differences turned out to be sex dependent (see Table 3). Females showed a large significant correlation between their valence and arousal ratings for especially the negative pictures with humans, while males showed significant correlations between their valence and arousal ratings for only the positive and neutral pictures independent of whether humans were depicted or not. This indicates that there are sex differences in the degree in which negative, positive and neutral pictures with and without humans are experienced as arousing. Matching pictures with and without humans with respect to valence or arousal (or even both) would therefore not only have been difficult but would also ignore the natural variation i.e. the individual differences in how the pictures are experienced. Moreover, assuming different neurophysiological systems underlying approach and avoidance behavior and thereby separability of the positive and negative motivational constructs, the bipolar valence ratings may not provide unequivocal information on how much positivity and how much negativity are activated [32]. This might be another reason for not matching the pictures on valence.

**Table 2:** Mean values and standard deviations of normative valence and arousal ratings of the pictures selected for the six categories (based on Lang, Bradley, & Cuthbert, 2008 [3]).

	Valence				Arousal			
Category (n)	All	Females	Males	♀ vs. ♂	All	Females	Males	♀ vs. ♂
	Mean (sd)	Mean (sd)	Mean (sd)	t	Mean (sd)	Mean (sd)	Mean (sd)	t
Humans (207)					4.77 (1.26)	4.87 (1.37)	4.62 (1.23)	4.8***
Negative (69)	2.26 (0.74)	1.96 (0.85)	2.63 (0.71)	−11.5***	5.91 (0.82)	6.18 (0.87)	5.59 (0.82)	10.5***
Neutral (69)	5.34 (0.62)	5.42 (0.77)	5.26 (0.52)	2.7**	3.54 (0.60)	3.59 (0.66)	3.51 (0.65)	ns
Positive (69)	7.13 (0.52)	7.38 (0.72)	6.77 (0.64)	5.5***	4.84 (0.97)	4.82 (1.03)	4.70 (1.12)	ns
Scenes (207)					4.26 (1.25)	4.27 (1.29)	4.21 (1.23)	ns
Negative (69)	3.49 (0.82)	3.13 (0.83)	3.92 (0.94)	−11.1***	5.44 (0.97)	5.57 (0.98)	5.28 (1.02)	5.0***
Neutral (69)	5.22 (0.69)	5.22 (0.75)	5.23 (0.67)	ns	3.26 (0.69)	3.23 (0.61)	3.28 (0.84)	ns
Positive (69)	6.92 (0.72)	7.16 (0.81)	6.65 (0.67)	9.4***	4.08 (0.91)	4.00 (0.91)	4.07 (0.90)	ns

T-values refer to a within category comparison of the normative male and female ratings.

Note: ***p<.001; **p<.01.

**Table 3:** Correlations between the pictures’ valence and arousal ratings.

Category (n*)	All	Females	Males
Humans (207)			
Negative (69)	−**.40 (p = .001)**	−**.44 (p<.001)^a^**	−.18 (p>.1)
Neutral (69)	.21 (p = .08)	.14 (p>.1)	**.41 (p<.001)^a^**
Positive (69)	.075 (p>.1)	.21 (p = .09)	**.51 (p<.001)^a^**
Scenes (207)			
Negative (69)	−.03 (p>.1)	−**.27 (p = .03)**	−.20 (p = .1)
Neutral (69)	**.42 (p<.001)**	**.25 (p = .04)**	**.56 (p<.001)^b^**
Positive (69)	.**24 (p = .05)**	.16 (p>.1)	**.42 (p<.001)^b^**

Note: *number of pictures; ^a/b^male and female correlations are significantly different (^a^one-sided; ^b^two-sided).

#### 2.2 Task

Participants were seated on a chair in front of a computer screen in a room separated from a control room by a one-way screen. They were instructed to focus on a small cross (2 mm) located in the centre of the screen and to avoid any body or eye movements. After a standardized instruction, they performed a short practice block of 5 minutes including only neutral pictures. The experiment was divided into three blocks of 161 pictures, each consisting of 138 photographs and 23 randomly inserted target stimuli (red-white squares) that required a manual response by pressing a button of a response panel. The targets were inserted to ensure attentive watching of the pictures. Within each block, the different categories were balanced and randomly presented. Between each block, a short break of a couple of minutes was given to the participants. The total duration of the session was about 45 minutes. The colored pictures with a size of about 29.5×24.5 cm were presented at the centre of an LCD screen (38.0×30.5 cm) against a black background with a viewing distance of about 70 cm. Presentation time was 1 s with a variable inter-stimulus interval (ISI) of 3 to 5 s. E-prime 2.0 was used to control presentation and timing of the stimuli.

### 3. Data collection and Preprocessing

#### 3.1 ECG recording and preprocessing of the ECR

While performing the task, the participants’ cardiac signal was recorded from pre-cordial leads by two Ag-AgCL electrodes placed at respectively the right side of the thorax beneath the collarbone and at the left side between the two lower ribs. A ground electrode was placed at the sternum.

The ECG was measured with a sample rate of 500 Hz. In order to analyze the ECRs, sequential Interbeat intervals (IBIs) were extracted from the R-peak series. R-peaks were detected online using Portilab (version 1.10, Twente Medical Systems International). To include only validly recorded IBIs, these were corrected for artifacts using the CARSPAN program for analysing cardiovascular data [33]. A procedure was adopted in which intervals that deviated more than four SDs from a running mean of 60 s were set as possible artefacts. Using a linear interpolation algorithm, corrections were made in case a set of additional criteria was met (for a more detailed description, see Mulder, 1992). Finally, all data were visually inspected in order to check for adequate corrections.

The IBI series was partitioned from 1 s before stimulus onset until 4.5 s after stimulus presentation. These 5.5 s data segments were re-sampled equidistantly with a frequency of 2 Hz resulting in 12 values measured at distances of half a second starting with two IBI values preceding the stimulus (i.e. IBIs at t = −1 and t = −0.5 s) followed by the IBI value corresponding with stimulus onset (IBI 0) and the nine IBIs after stimulus onset. A step size of half a second was chosen to display the (brief) alterations in IBI values accurately. All IBI values up from time points −0.5 until 4.5 s were adjusted for a baseline to make individuals comparable with respect to their relative picture condition-dependent changes in IBI. The baseline value was the mean IBI at t = −1 s, which was subtracted from each of the succeeding values in the data segment. From these baseline-adjusted values the individual’s deceleration maxima were determined in the cardiac evoked response curve running from −0.5 s to 4 s (IBI_MAX_). These values were analyzed as ECRs to the various picture conditions.

#### 3.2 EEG recording and pre-processing of the LPP

**Figure 1:**
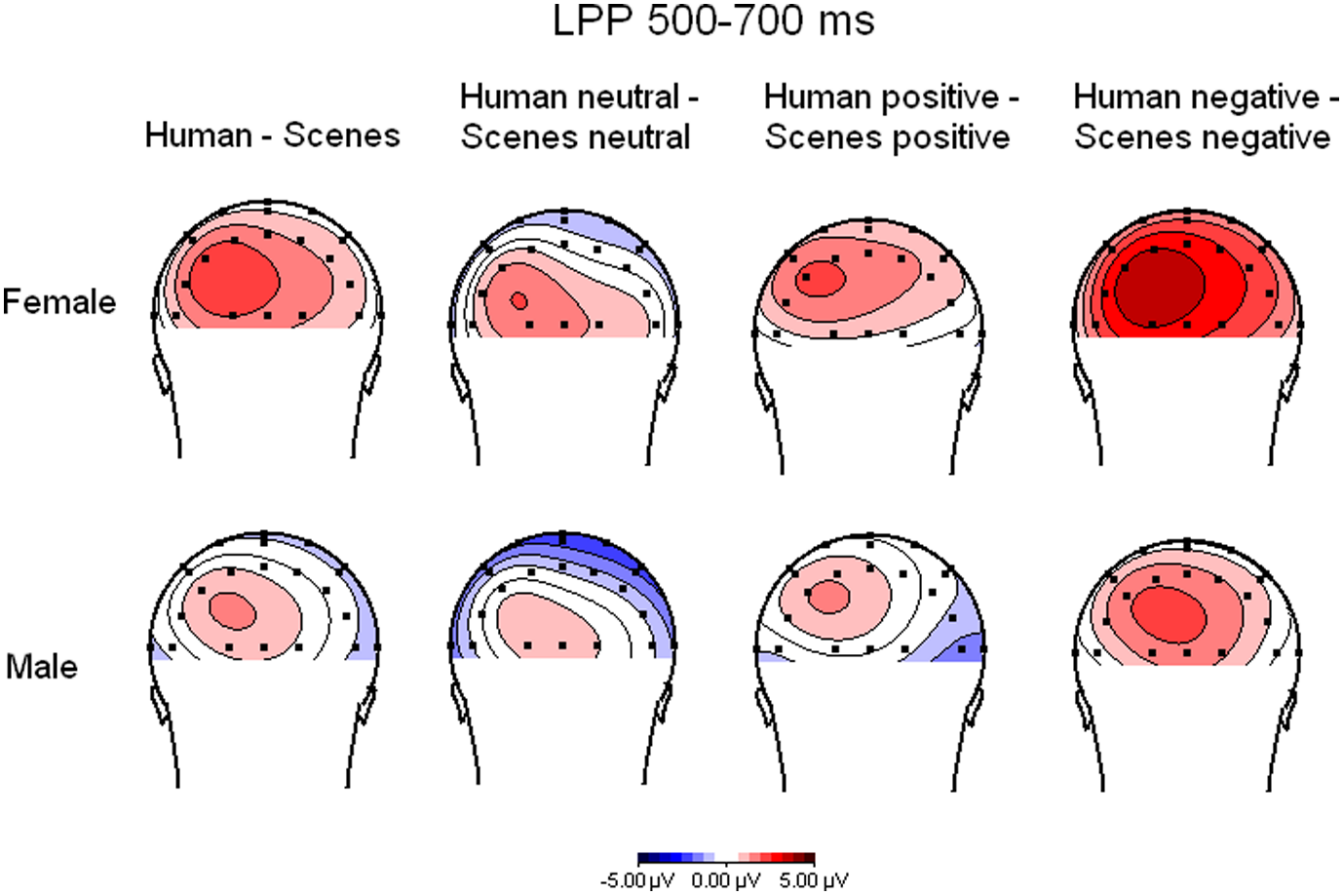
Topographical maps of the difference waves of ERPs in response to pictures with and without humans independent of valence (most left), neutral pictures with humans minus neutral pictures without humans, positive pictures with humans minus positive pictures without humans, and negative pictures with humans minus negative pictures without humans (most right).

As the LPP data used for the comparisons made in the present paper are the same as those described in the paper by Groen and colleagues (2013) we refer to that paper for a complete description of the EEG recording (http://dx.doi.org/10.1016/j.neuropsychologia). Note that the number of participants for the ERP analyses was smaller (n = 52; 25 women) than for the ECR analysis since participants had to be omitted due to EEG artifacts caused by a technical failure. Here, we will briefly summarize how the choice of the electrode positions and time windows was made, based on among others topographical maps. Visual inspection indicated that the LPP reached its maximum between 500–700 ms on occipito-parietal electrode positions. Difference waves of the potentials for humans minus scenes indicated that the largest human effects were present over left occipito-parietal (for negative pictures with humans) and parietal (for positive pictures with humans) electrode positions (see Figure 1). To obtain a precise indication of the time course of the on- and offset of effects, the LPP was previously quantified as the average amplitudes on P3, P4, P7, and P8 in successive time intervals of 50 ms in the time window of 400–800 ms (see Table 5 in Groen et. al., 2013).

For the comparisons made in the present study we quantified the LPP as the average amplitude of the three 50 ms intervals running from 550 ms to 700 ms because in these intervals most effects were found. We will report the results for only the P3 and P7 electrode positions as human effects were found to be largest for the left hemisphere (Figure 1).

### 4. Statistical Analyses

#### 4.1 Task effects and Sex differences on ECR parameters

To test our first two hypotheses, cardiac responses were analyzed by means of repeated measures analysis of variance (IBM SPSS 19, GLM, repeated measures). Analyzed were the individuals’ baseline-adjusted deceleration maxima (IBI_MAX_). We used a 2*3*2 ANOVA design with one between subject variable, i.e., Sex, and two within-subject variables, i.e., the presence of Humans (humans vs. scenes) and the Valence condition (positive vs. negative vs. neutral). For effects with Valence involved, Greenhouse-Geisser adjusted p-values and the epsilon correction factors are reported. Planned comparisons were carried out by computing contrasts between each of the three valence pairs.

For all comparisons, next to the p-values, Cohen’s *d* or partial eta squared are presented as measures of effect size, with *d*<0.5 and *η^2^*<.06 reflecting a small effect; *d*≥0.5 and *η^2^*≥.06 a medium effect; and d≥0.8 and *η^2^*≥.14 a large effect.

#### 4.2 Task effects and Sex differences on the LPP values

LPP values were analyzed according to the same 2*3*2 ANOVA design as used for the cardiac IBI_Max._ Separate analyses were carried out for the mean amplitudes of the P3 and P7 position.

#### 4.3 Comparison of the effects on the ECR and LPP

The (sex differential) task effects on the ECR were compared to those found for the LPP in terms of their presence, direction, and effect sizes. Similarities and differences of the two measures are visualized by graphs depicting the means and SEMs of the different task conditions for men and women separately. Moreover, logistic regression analysis was conducted to investigate the relative contribution of the task effects on ECR and LPP in predicting sex differences.

#### 4.4 Associations between ECR, LPP and questionnaire measures

To explore whether the ECR and LPP responses to affective pictures with humans correlate with the questionnaire scales assessing empathy-related traits, we first computed Pearson’s correlations between the questionnaire scale scores and the task effect dependent difference scores of the IBI_Max_ and LPP, respectively (ΔIBI_Max_/ΔLPP). To this end, the main effects of (1) Human (Hum) (2) Valence (Val), and (3) the interaction effect of Human*Valence were translated into the following effect scores (delta values).

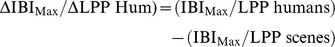

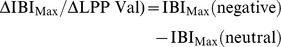

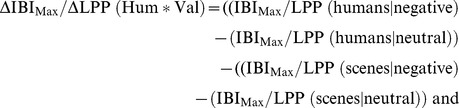

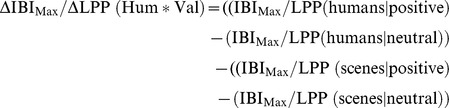

Pearson correlations were computed between these delta values and the subscale scores of the IRI, the EQ score and the SQ–EQ discrepancy score. Also, correlations were investigated between the ECR and LPP in terms of their sensitivity to (valence dependent) Human effects.

Finally, to reduce the number of statistical tests and to investigate whether (valence dependent) Human effects on the LPP and ECR might form a significant linear combination in the prediction of self-reported empathy, we conducted stepwise multiple regression analyses using the effect measures computed for both the ECR and LPP as predictor variables and those empathy-related scales as dependent variables that turned out to correlate significantly with (one of the) the effect measures on a single test level.

## Results

### 1. Evoked Cardiac Response

#### 1.1 Task manipulation effects

**Figure 2:**
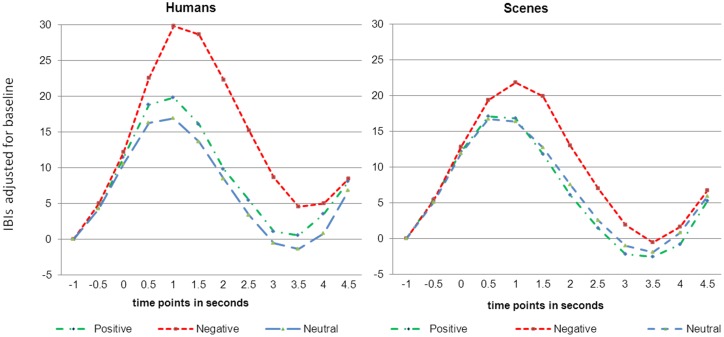
Baseline-adjusted IBIs (in milliseconds) in response to the IAPS pictures (t = 0 s). Left panel: pictures with humans present; right panel: pictures without humans.

**Figure 3:**
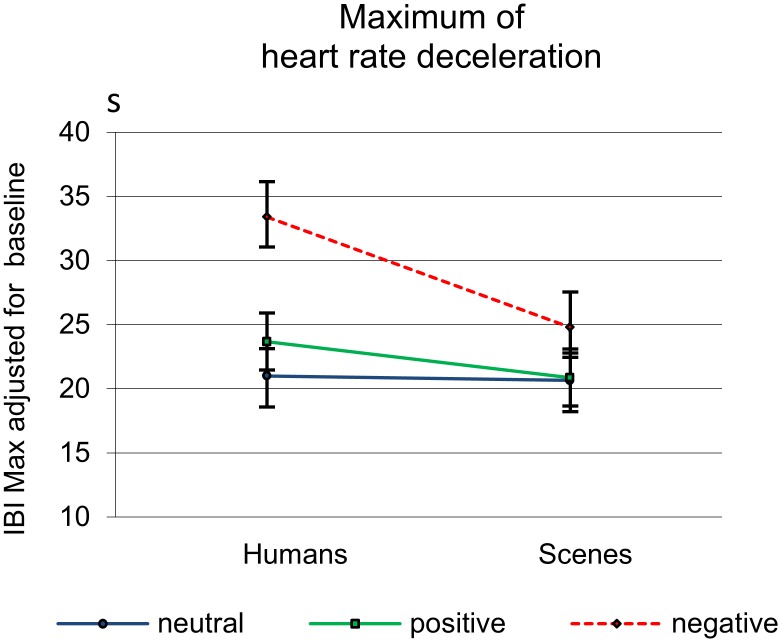
Means and SEMs of the baseline-adjusted maximal IBIs (in milliseconds) illustrating the Human by Valence effect. Note that these maxima are not completely equal to those of the deceleration curves as IBI_max_ is based on the individuals’ maxima, which may differ in delay.

Figure 2 shows that the general decelerative response pattern starts to differentiate for the emotional valence of the stimulus from half a second after picture presentation. Note that an IBI increase corresponds to a heart rate decrease. The deceleration is steepest at about 1 s after picture presentation and then gradually returns to baseline. Effects for IBI_Max_ are summarized in Table 4. Significantly greater responses to pictures with humans than to pictures without humans were found (see also Figure 2). There were also valence effects, but these held for only the contrasts with the negative pictures. There were no differences in response to positive vs. neutral pictures. Moreover, valence interacted significantly with presence of humans. This interaction, however, held again for only the contrast with negative pictures. Figure 3 shows that the interactive effect of Human and Valence is due to in particular the value for negative pictures with humans. When testing human effects for the different valences separately, baseline-adjusted IBI_Max_ in response to negative pictures with humans turned out to differ significantly from the response to negative pictures without humans (*p*<.001; *d* = 0.54), So did IBI_Max_ in response to positive pictures with humans as compared to IBI_Max_ in response to positive pictures without humans (*p* = .025; *d* = 0.24). There were no differential heart rate responses to neutral pictures with and without humans. Our first hypotheses that affective pictures with humans, especially when being aversive, should elicit greater cardiac evoked responses than pictures without humans could hence be confirmed.

**Table 4:** ANOVA effects for the task variables Human and Valence and their interaction with Sex on the maxima of the baseline-adjusted IBIs.

IBI_Max_	df	F	p	ε	η^2^
Human	1,62	11.31	.001	-	.15
Valence	2,124	18.92	<.001	.96	.38
negative>neutral	1,62	37.10	<.001	-	.37
negative>positive	1,62	17.96	<.001	-	.23
Human*Valence	2,124	6.41	.004	.96	.17
negative>neutral	1,62	11.12	.001	-	.15
negative>positive	1,62	6.44	.014	-	.09
Sex*Human	1,62	5.96	.017	-	.08
Sex*Valence	2,124	-	>.1	-	-
Sex*Human*Valence	2,124	-	>.1	-	-

#### 1.2 Sex differences

Sex differential effects are also presented in Table 4. There were no Sex*Valence and Sex* Human*Valence interaction effects. Yet, men and women did significantly differ in their responses to the presence vs. absence of humans as is reflected in the two-way interaction Sex*Human (Table 4).

**Figure 4:**
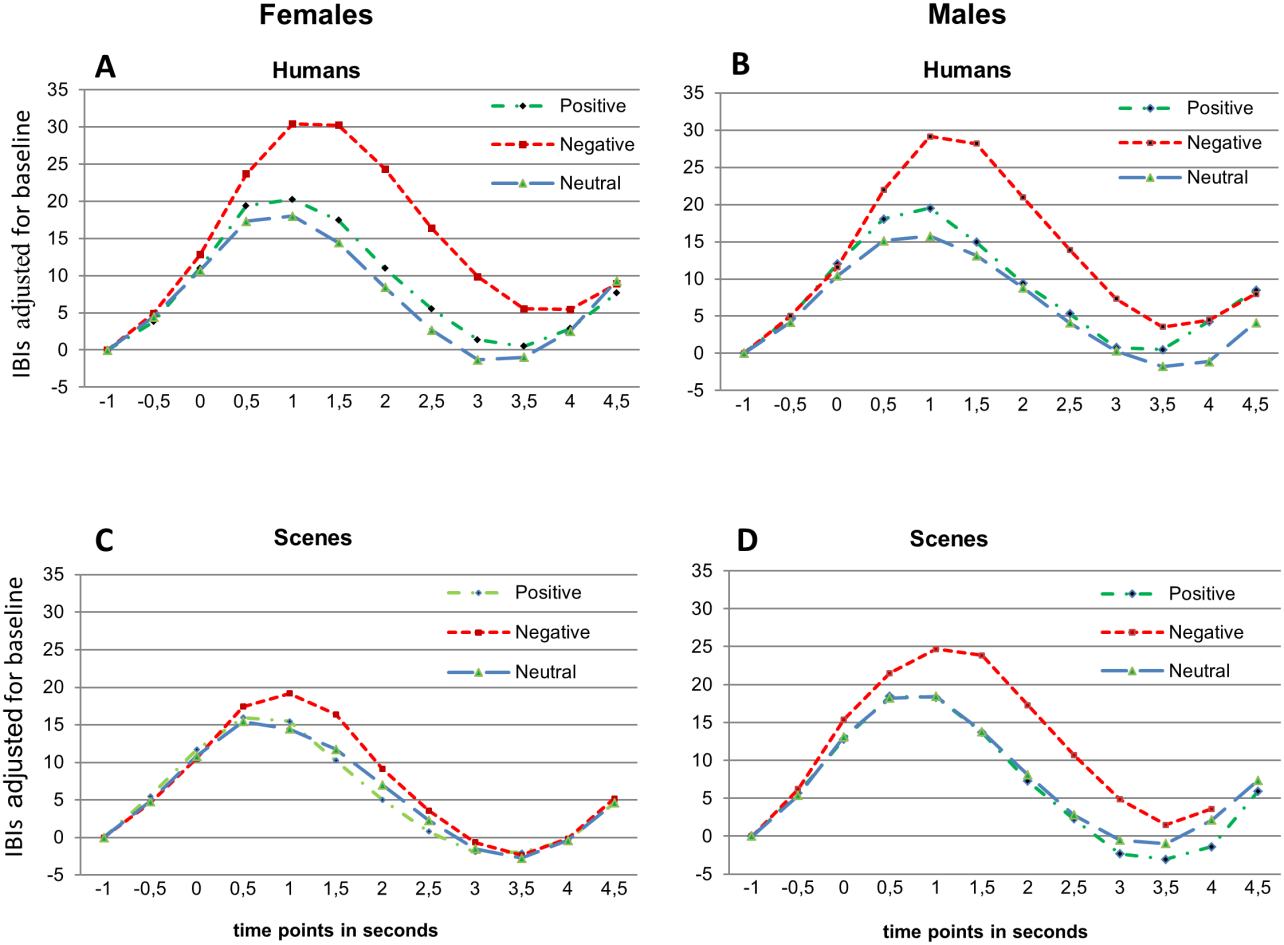
Baseline-adjusted IBIs in response to the IAPS pictures (t = 0 s) separated per sex. Upper panel: humans present in the pictures; lower panel humans absent.

**Figure 5:**
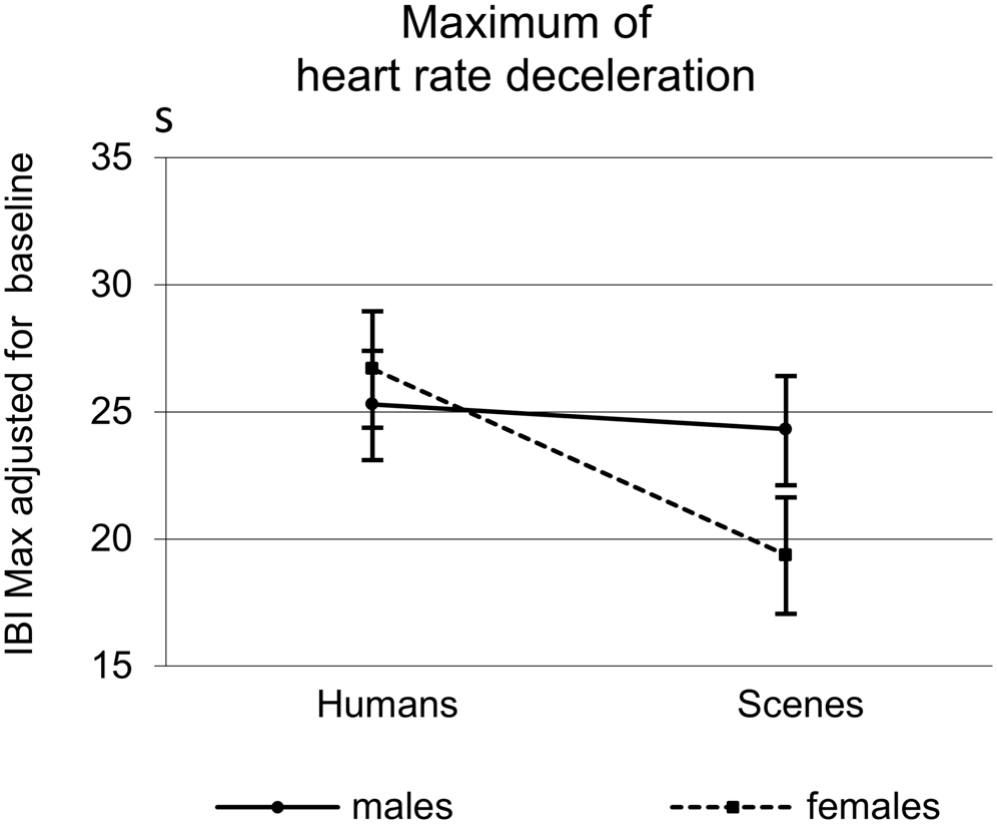
Means and SEMs of the baseline-adjusted maximal IBIs illustrating the sex differential Human effect.

However, different from what has been hypothesized, men and women did not differ in their responses to pictures with humans (Figure 4a and 4b) but in their responses to pictures without humans, with men showing the greater response (Figures 4c and 4d). While no significant difference was found between the male cardiac responses to pictures with and without humans, women showed a significantly smaller deceleration maximum to pictures without humans as compared to those with humans (*t* = 4.852; *df* = 33; *p*<.001; *d* = 0.85), which is illustrated in Figure 5.

### 2. Comparison of the ECR with the LPP

#### 2.1 Task effects

**Figure 6:**
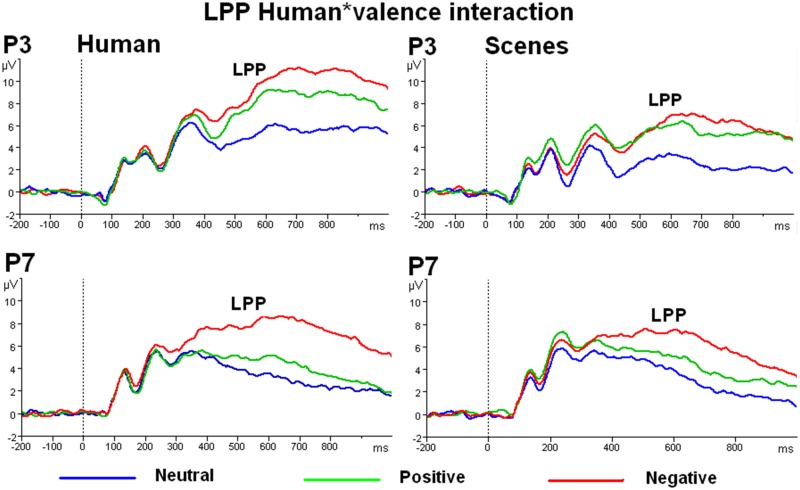
ERPs in response to pictures with and without humans illustrating the Human (P3) and Human*Valence interaction (P7) effects of the LPP in the time window of 550 to 700 ms.

Similar to the ECR deceleration, a large Human effect was found for the LPP on P3 (see Table 5). Also similar to the ECR, for the LPP on P7 the largest response was found for negative human emotions (Table 5 and Figure 6).

**Table 5:** ANOVA effects of the task variables Human and Valence and their interaction with sex on the LPP mean amplitude of the interval 550 to 700 ms after stimulus presentation.

LPP 550–700 ms	P3					P7				
	Df	F	p	ε	η^2^	df	F	p	ε	η^2^
**Human**	1,50	85.57	<.001	-	.63	1,50	-	>.1	-	-
**Valence**	2,100	64.65	<.001	.85	.56	2,100	80.15	<.001	.83	.62
** negative>neutral**	1,50	90.85	<.001	-	.65	1,50	110.1	<.001	-	.69
** negative>positive**	1,50	9.28	.004	-	.16	1,50	71.17	<.001	-	.59
** positive>neutral**	1,50	102.15	<.001	-	.67	1,50	27.03	<.001	-	.35
Human*Valence	1,50	-	>.1	.96	-	1,50	5.07	.008	.90	.09
** negative>neutral**	1,50	-	>.1	-	-	1,50	7.78	.007	-	.14
** negative>positive**	1,50	-	>.1	-	-	1,50	9.81	.003	-	.16
Sex*Human	1,50	5.73	.02	-	.10	1,50	7.16	.01	-	.13
Sex*Valence	2,100	3.72	.04	.85	.07	2,100	3.35	.05	.83	.07
** negative>neutral**	1,50	4.24	.05	-	.08	1,50	-	>.1	-	-
** negative>positive**	1,50	5.25	.03	-	.10	1,50	6.7	.013	-	.12
Sex*Human*Valence	2,100	-	>.1	-	-	2,100	-	>.1	-	-

#### 2.2 Sex differences

**Figure 7:**
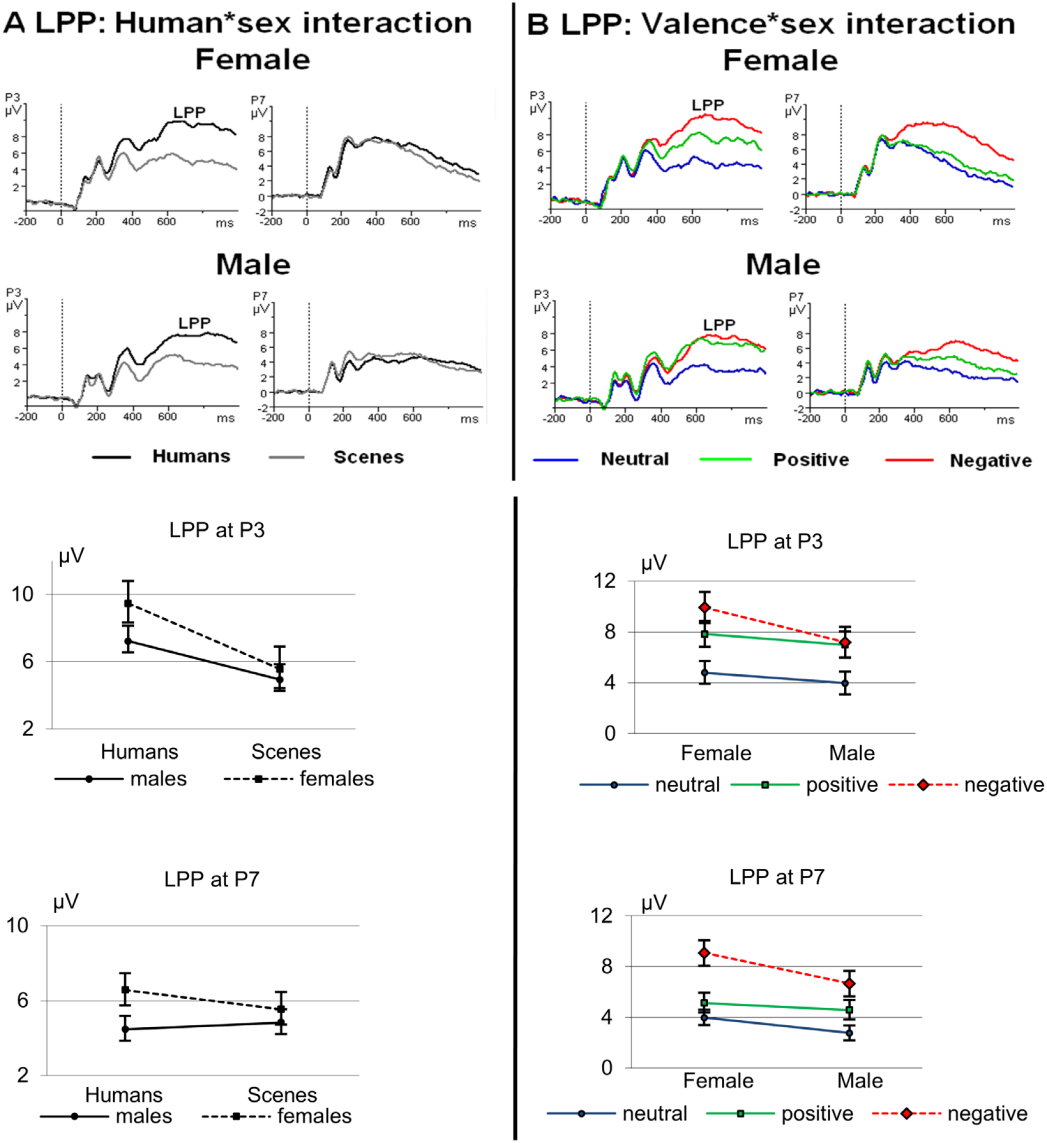
ERPs of parietal electrodes (P3 and P7) depicting the Human*Sex and the Valence*Sex interaction. Below the ERPs showing the grand averages for pictures with and without humans collapsed for valence (A) and for pictures with the different valences collapsed for human presence (B). The corresponding line graphs with mean amplitudes (and SEMs) of the 550 to 700 ms interval illustrate the interactions found.

While at the P3 position both men and women showed a significantly larger LPP to pictures with humans as compared to pictures without humans (men: *t* = 4.79; *df* = 26; *p*<.001; *d* = 1.01; women: *t* = 8.38; *df* = 24; *p*<.001; *d* = 1.82), this difference was smaller in men than in women leading to a significant Sex*Human interaction (Table 5; Figure 7A, left upper and first lower panels). For the LPP at P3, in both men and women, the Human effect held for all valences. Quite similar to the ECR, at electrode position P7, men did not show any significant difference in their responses to pictures with and without humans at all, while women did (*t* = 2.88; *df* = 24; *p* = .008; *d* = 0.59), which also resulted in a significant Sex*Human interaction (Table 5, Figure 7A, right upper and last lower panels).

**Figure 8:**
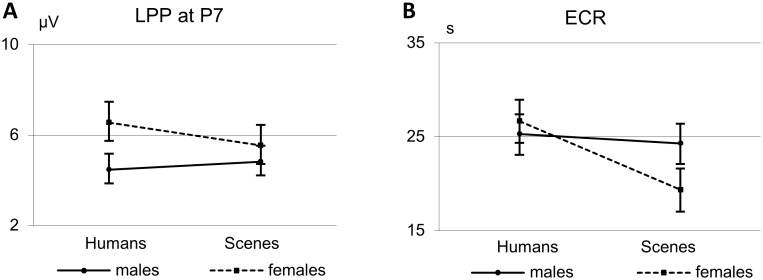
Means and SEMs of the LPP values (average amplitudesof the 550 to 700 ms interval) at P7 (a) and the baseline-adjusted maximal IBIs (b) illustrating the sex differential Human effects.

Yet, when further analyzing the Sex*Human interactions found for the LPPs on the one hand and the ECR on the other with respect to sex differences in response to pictures with humans and pictures without humans separately, a remarkable difference emerged. While the LPP, especially at P7, discriminated men and women in their response to pictures *with* humans, (women showing the greater LPP, *t* = 2.675; *p* = .01; *d* = 0.76), the ECR (IBI_Max_) discriminated men and woman in their responses to pictures *without* humans, (men showing the greater deceleration response, *t* = −2.44; *p* = .018; *d* = 0.61) (Figure 8).

Also different from the ECR, Sex*Valence effects were found for both the P3 and P7 with a significantly greater female responsiveness to negative pictures as compared to positive and neutral ones (Table 5, Figure 7B, lower panels). No such interaction was found for the ECR (see Figure 4).

#### 2.3 Prediction of Sex by Human effects on the LPP and ECR

Logistic regression with sex regressed upon both the Human effect on the LPP and the Human effect on the ECR was carried out with the mean amplitudes of the LPP at the P7 position as this was the position showing the larger Human*Sex interaction. Forced entry showed that the LPP was entered with *p* = .019 (*Wald* = 5.481; *df* = 1) and the ECR with *p* = .075 (*Wald* = 3.17; *df*  = 1). The chi-square for the omnibus test of model coefficients increased from *χ* ^2^ = 5.75; *df* = 1; *p* = .016 (LPP only) to *χ* ^2^ = 9.51; *df* = 2; *p* = .009 (LPP and ECR). With both predictors in the equation the percentage of correctly classified individuals enhanced from 58% (LPP only) to 68% (LPP and ECR), the percentage being equally large for men and women. Hence, including the ECR improved the prediction of sex differences in orienting to social information.

### 3. Correlations between ECR, LPP and questionnaire measures

There were no significant correlations between the (valence dependent) Human effects on the ECR and LPP. On a test-wise level, there was a significant correlation between the human negative emotion effect on heart rate slowing and one of the two self-report measures assessing *affective* empathy i.e. the IRI EC scale (see Table 6). Moreover, the effect of humans being present in the pictures on the LPP at both P3 and P7 appeared to correlate with the other scale measuring affective empathy, i.e. the IRI PD scale. Finally we found significant negative correlations between the Human effect on both the ECR and LPP at the P3 position with the SQ-EQ discrepancy score suggesting that relatively more systemizing as compared to empathizing qualities go along with smaller orienting responses to pictures with humans as compared to pictures without humans. Note, that no correlations were found with any of the scales measuring cognitive empathy, nor with the total EQ score.

**Table 6:** Correlations (Pearson’ s r) of the human effects on the maximal IBIs adjusted for baseline and the human effects on the LPP components with the questionnaire scales measuring various aspects of empathy: IRI PT: perspective taking; FS: fantasy; EC: empathic concern; PD: personal distress; EQ: empathy quotient and D_SQ-EQ: the discrepancy between systemizing and empathizing behavior.

Effects on			IRI PT	IRI FS	IRI EC	IRI PD	EQ	D_SQ-EQ
IBI_Max_ a	Human	r	.090	−.17	−.117	−.035	.089	−**.339**
		p	>.1	>.1	>.1	>.1	>.1	**.02**
		n	63	63	63	63	63	**47**
	Human*Valence^b^	r	−.049	.11	**.279**	.005	.102	−.007
		p	>.1	>.1	**.018**	>.1	>.1	>.1
		n	63	63	**63**	63	63	47
LPP P3^c^	Human	r	−.16	.026	.268	**.393**	.10	−**.367**
		p	>.1	>.1	.07	**.005**	>.1	**.018**
		n	50	50	50	**50**	50	**41**
	Human*Valence^b^	r	−.141	.058	.065	.237	−.111	−.040
		p	>.1	>.1	>.1	.09	>.1	>.1
		n	50	50	50	50	50	41
LPP P7^c^	Human	r	−.196	.04	.059	**.292**	−.039	−.201
		p	>.1	>.1	>.1	**.04**	>.1	>.1
		n	50	50	50	**50**	50	41
	Human*Valence^b^	r	−.036	.058	.24	**.291**	−.043	−.049
		p	>.1	>.1	.09	**.04**	>.1	>.1
		n	50	50	50	**50**	50	41

IRI EC and IRI PD are measures of affective empathy.

a one subject was omitted as outlier (z- value on effect measure < −3);

b interaction refers to the contrast negative vs. neutral only, as the contrast positive vs. neutral was not significant;

c two subjects were omitted as outliers; significant correlations are printed bold.

In order to prevent capitalizing on chance, we confined the number of statistical tests while carrying out three stepwise multiple regression analyses, regressing the three empathy-related measures that showed any association with the orienting responses at a test-wise level i.e. the IRI EC, IRI PD and D_SQ-EQ scores upon the ECR and LPP responses to (negative) pictures with humans. As more and greater correlations were found for the LPP at the P3 than at the P7 position we chose to only enter the LPP P3 responses in combination with the ECR responses as predictor variables. In a next step we entered the interaction terms Sex*LPP/ECR response in the model in order to investigate moderator effects of sex. This lead to 2*3 models compared, with a required statistical significance level of α ≤.008 (Bonferroni-corrected). In case the sex-interactions contributed significantly, post hoc regression analyses were carried out for men and women separately.

Table 7 summarizes the results of the multiple regression analyses. Four of the six models were statistically significant (p<.008). Regressing the **IRI EC** scale upon the Human and Human*Valence (negative) effect on the two orienting responses resulted in none of the responses being included as a predictor of empathic concern. Also, adding the sex interaction term did not result in a significant prediction of IRI EC.

**Table 7:** IRI Empathic Concern, IRI Personal Distress and Systemizing-Empathizing discrepancy scores regressed upon Human and Human*Valence effects on IBI_max_ and LPP at the P3electrode position.

ECR and LPP at P3 Stepwise entered	IRI EC		IRI PD		D_SQ_EQ	
	β	p	β	p	β	p
IBI_Max_ (Human)	-	-	-	-	−**0.31**	**.04**
IBI_Max_ (Human by Valence)	-	-	-	-	-	-
LPP P3 (Human)	-	-	**0.39**	**.005**	−**0.37**	**.01**
LPP P3 (Human by Valence)	-	-	-	-	-	-
	No variables entered		**R^2^ = .155**		**R^2^ = .189**	
	F(4,45) = 1.04; p = .39		**F(1,48) = 8.78; p = .005**		**F(2,38) = 5.65; p = .007**	
With sex interactions added	β	p	β	p	β	p
Sex * IBI_Max_ (Human)	-	-	-	-	-	-
Sex * IBI_Max_ (Human by Valence)	-	-	-	-	-	-
Sex * LPP P3 (Human)	0.28	.05	**0.439**	**.001**	−**1.479**	**.001**
Sex * LPP P3 (Human by Valence)	-	-	**0.294**	**.02**	-	-
	R^2^ = .079		**R^2^ = .272**		**R^2^ = .293**	
	F(1,48) = 4.09; p = .05		**F(2,47) = 8.76; p = .001**		**F(1,39) = 16.19; p<.001**	
Men						
LPP P3 (Human)	.26	>.1	**0.49**	**.01**	0.25	>.1
LPP P3 (Human by Valence)	-	-	0.19	>.1	-	-
Women						
LPP P3 (Human)	.12	>.1	0.15	>.1	−**0.48**	**.03**
LPP P3 (Human by Valence)	-	-	0.30	>.1	-	-

**Figure 9:**
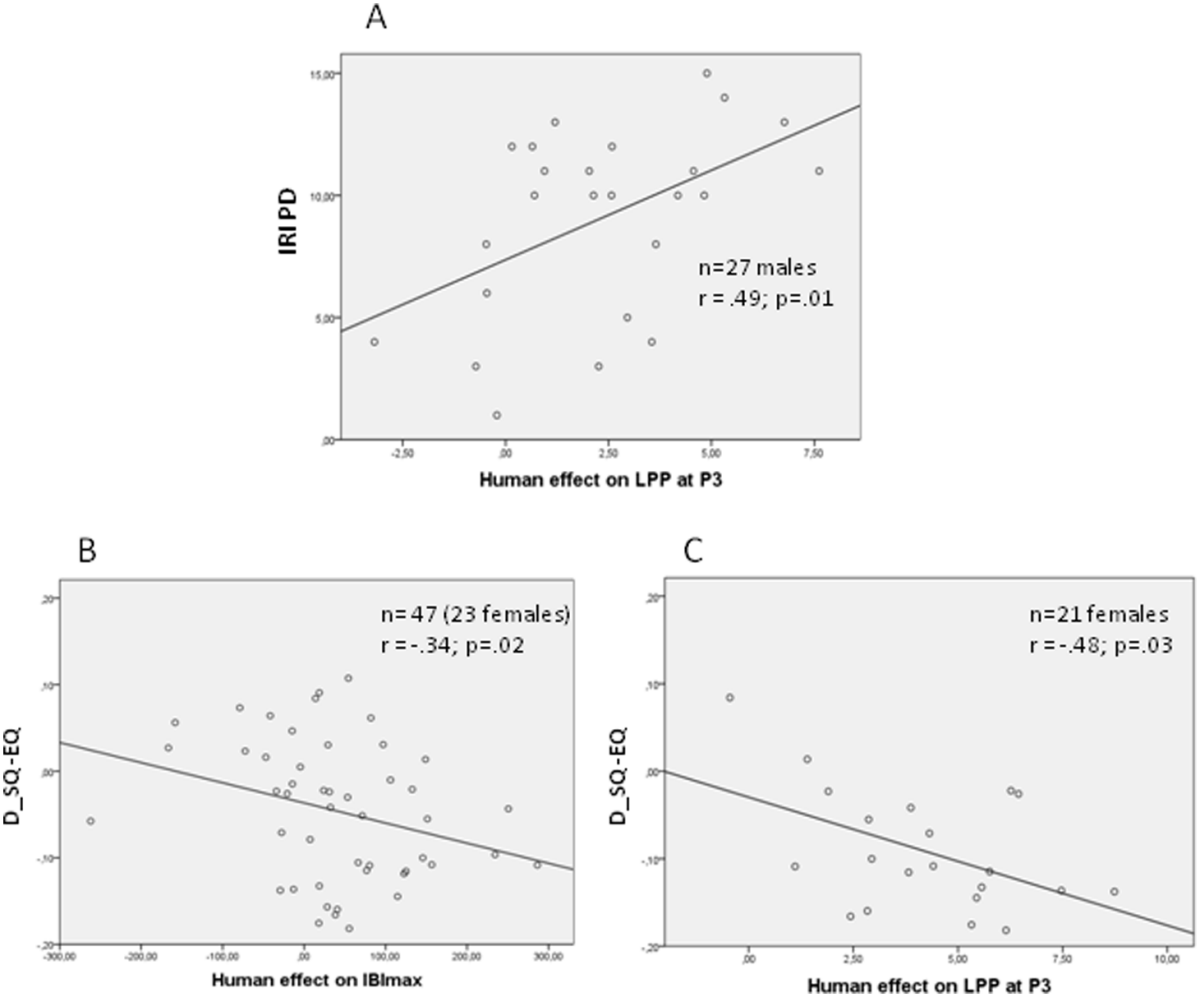
Scatter plots for the correlations between (A) the Human effect on the LPP at P3 and the Personal Distress scale of the IRI for the males only, (B) the Human effect on the baseline-adjusted maximal IBI’s and the discrepancy between systemizing and empathizing behavior (D_SQ-EQ) for all participants, and (C) the Human effect on the LPP at P3 and the D_SQ-EQ for the females only.

Regressing the **IRI PD** scale upon the Human and Human*Valence affected orienting responses resulted in the inclusion of the Human effect on the LPP as a significant predictor. Taking into account the moderating effect of sex, we found the prediction of IRI PD by the LPP significant for the men only (r = .49), reflecting that larger IRI PD scores are associated with larger responses to pictures with humans as compared to pictures without humans (Figure 9A).

The Systemizing-Empathizing discrepancy score (**D_SQ_EQ**) could be predicted by the Human effect on the LPP and the Human effect on IBI_max_. For both, the unstandardized regression coefficients differed significantly from zero, and semi-partial correlation coefficients for the LPP and ECR (*r* = −.37 and *r* = −.33, respectively) show a similar unique portion of variance explained in the discrepancy between self-reported systemizing and empathizing behavior. The prediction by the LPP was moderated by sex, apparently holding for only the women (r = −.48). In both cases (IBI_max_ and LPP), relatively more systemizing as compared to empathizing behavior appeared to be associated with a smaller difference between responses to pictures with and without humans (Figures 9B and 9C).

## Discussion

### 1. Summary of the main findings

The comparative part of our study showed similarities and differences in sensitivity of the LPP and ECR to the presence of humans in the pictures as well as to the pictures’ valence. As hypothesized, both types of responses were significantly larger when humans were portrayed and largest when the pictures with humans had a negative valence. Differences emerged when sex was taken into account. While for the LPP there was a valence by sex interaction, women showing a larger response than men to especially the unpleasant pictures, no such interaction was found for the ECR. Moreover, although there were significant human by sex interactions for both types of responses, the direction of this interaction had a different appearance for the LPP than for the ECR: while women showed a greater LPP response than men to pictures with humans, men showed a greater ECR response than women to pictures without humans.

The explorative part on brain behavior correlations showed that both types of responses were associated with empathy-related self reports. While the LPP response to pictures with humans showed a positive correlation with the Personal Distress (PD) scale of the IRI, particularly in men, as well as a negative correlation with the discrepancy measure describing more systemizing as compared to empathizing behavior, particularly in women, the ECR in response to pictures with humans showed a negative correlation with the systemizing/empathizing discrepancy measure only. For both responses relatively more systemizing as compared to empathizing behavior was associated with a smaller difference between responses to pictures with and without humans.

### 2. Sex differences in self-report measures and orienting responses

Our study could replicate the findings of several previous studies reporting higher female scores in self-report measures of empathy [13]–[15]. No sex difference, however, was found in the specific component of cognitive empathy as is assumed to be measured by the Perspective Taking (PT) scale of the IRI. This may be due to both men and women in our study being students of psychology who share a generally enhanced interest in other people. Cognitive empathy is supposed to develop later in life than affective empathy and to be much more susceptible to learning [34].

The finding of our female students reporting greater personal distress than men when experiencing others in distress (IRI PD scale) is in line with women having judged the aversive pictures with humans as more arousing than men as the IRI PD scale has previously been found to be related to greater emotionality, vulnerability and fearfulness [14].

Our cardiac findings, however, do not agree with these subjective reports; women did not show greater cardiac reactivity than men to aversive pictures with humans. Moreover, and this differs from what has been found for the LPP, even the *overall* greater decelerative ECR response to unpleasant pictures was the same for men and women. Our heart rate findings, therefore, do not support the hypothesis of women having a generally more sensitive avoidance system than men, while the LPP findings might support this hypothesis.

For both measures, the ECR and LPP, greater differences in the responses to pictures with and without humans were found for women than for men. However, these sex-differential responses to pictures with and without humans turned out to have different appearances. While the LPP distinguishes men and women in their responses to pictures *with* humans (women showing the greater response) the ECR distinguishes them in their responses to pictures *without* humans (men showing the greater response) (Figure 9). In other words, as compared to the other sex, women showed a greater electrocortical orientation to social scenes, while men showed greater autonomic cardiac orientation to nonsocial scenes. The equally strong cardiac orienting to social and nonsocial scenes in our group of male students suggests that men may experience the two types of scenes as equally relevant. This is in contrast to the significant difference in their subjective arousal ratings of scenes with and without humans and suggests that the vagally controlled ECR might be less affected by arousal than their subjective experience of the pictures.

Here we will speculate on some phylogenetic explanation. Slowing of the heart rate has previously been described as being anticipatory to an effective motor performance, especially in case the organism has to engage in a defense reaction [29]. Considering that the amygdala is implicated in the reflexive and unconscious responding to salient and biologically relevant stimuli [36], the defense preparatory role of the cardiac orienting response is further supported by the finding that the amygdala plays a role in the central regulation of heart rate during the processing of aversive information [37]. Hence in males, a greater deceleration response to (especially unpleasant) nonsocial scenes might reflect the organism’s preparation for a potential defense reaction, i.e. a fight or flight response.

The differential contribution of the two orienting responses to the prediction of sex has been supported by logistic regression analysis showing an acceptable percentage of correctly classified men and women only when both responses were entered in the equation as predictor variables. The sex differential pattern of the LPP reflecting women’s greater response to social scenes and the ECR reflecting men’s greater (defense preparatory) response to non-social scenes might support the evolutionary view on the propagation of mammalian species supposing the female gender being responsible for “tending and befriending” and the male gender for “defending” [38]. The stronger cardiac orienting to nonsocial information in males than in females might also be in line with the male brain hypothesis proposing the more systemizing male brain to be more prepared for the processing of nonsocial events and to have a greater need for a predictable environment [39].

### 3. Associations of the orienting responses with empathy-related self-reports

Only the enhanced LPP to pictures with humans appeared to be correlated with the IRI PD scale assessing discomfort when experiencing others in distress. As the IRI PD scale has been found to be related to greater emotionality and fearfulness [14], this correlation may suggest that the LPP is more affected by an arousal-related, i.e. sympathetically controlled sensitivity to affective information than the vagally controlled decelerative ECR.

The effect of social relevance (Human effect) on both the LPP (at the P3 position) and the cardiac response correlated significantly and negative with the discrepancy between self-reported systemizing and empathizing behavior, indicating that a more empathizing brain goes along with both an increased cardiac and electrocortical orienting to socially relevant pictures. Here it is important to stress that the two types of orienting responses could be shown to have a *complementary* contribution to the prediction of the systemizing-empathizing (S-E) discrepancy while explaining an almost equally large percentage of variance. The finding that the LPP correlation turned out to hold for the women in particular means that women with smaller responses to pictures with humans have a relatively stronger systemizing brain than those with larger LPP responses to pictures with humans. The previously proposed idea of a relatively stronger cardiac response to nonsocial information going along with a more systemizing brain is not only supported by the male students having reported significantly larger SQ and SQ-EQ discrepancy scores but also by the negative correlation of the SQ-EQ discrepancy score with the difference in both LPP and ECR to pictures with and without humans.

Based on the S-E discrepancy score, the different brain types that had previously been identified were shown to be associated not only with sex, more men than women having a systemizing brain, but also with autism, an *extremely* systemizing brain characterizing many autistic people. This latter phenomenon has resulted in the “extreme male brain theory of autism” outlined by Baron-Cohen and colleagues [40]. Using both types of orienting responses to socially relevant information might be valuable in identifying and distinguishing pathological conditions related to social behavior deficiencies.

### 4. Conclusions and future research

Both the ECR and LPP were larger to pictures with humans as compared to pictures without humans, and both these responses appeared to correlate with a measure of a more empathizing than systemizing coping style. Differences emerged when comparing the two orienting responses with respect to their sex dependency. While the LPP showed greater female than male susceptibility to the presence of humans in the pictures, the ECR showed a greater male than female response to the pictures without humans. The sensitivity of the two orienting responses to the relevance of social information and their differential contribution to the prediction of individual differences point to common as well as unshared origins underlining the validity of their combined use in clinical studies investigating individuals with social disabilities.

### 5. Limitations of the study

Our sample consisted of only psychology students. This confines the generalizability of our findings as sex differences in empathic capacities might be smaller in such a group than in the general population, due to the participants’ common enhanced interest in people. Less data were available for the LPP than for the ECR analyses. Yet, as the effects and correlations found for both measures were quite similar, not only in their statistical significance but also with regard to their magnitude, we think that this difference in sample size did not affect their comparability. Furthermore, less subjects completed the systemizing questionnaire. Nevertheless correlations were found between the systemizing/empathizing discrepancy score and the orienting responses to pictures with as compared to those without humans. These might have been stronger with more subjects having completed the questionnaire.

In the present study the pleasant and unpleasant pictures were not balanced in terms of subjectively experienced intensity and arousal. The reason for this was that there are sex differences in the experienced valence and arousal levels of these pictures (see section 2.2.1). These individual differences in subjective ratings make it impossible to match on these variables without taking away the sex-inherent differences in both subjective ratings and physiological responsiveness. Moreover, the negative pictures portraying people were experienced i.e. subjectively rated as more negative and more arousing than negative pictures without humans. This could be interpreted as a confounder. Yet, although there may be individual differences, the presence of humans in unpleasant pictures obviously reinforces the subjective feeling of unpleasantness and arousal [31], and the fact that stimuli depicting humans are experienced as more arousing is likely part of the empathic experience. We therefore believe that matching affective pictures with humans to those without humans in terms of valence and arousal would eliminate the most empathy-evoking pictures. The higher arousal and unpleasantness experience of pictures with humans may therefore not be a confounding factor but rather a mediating factor in the experience of empathy. Finally, as could be shown in the present study, subjective arousal ratings may differ from physiological orienting to affective information.
